# A Pilot Study of Possible Easy-to-Use Electrophysiological Index for Early Detection of Antidepressive Treatment Non-Response

**DOI:** 10.3389/fpsyt.2017.00128

**Published:** 2017-07-18

**Authors:** Goded Shahaf, Shahak Yariv, Boaz Bloch, Uri Nitzan, Aviv Segev, Alon Reshef, Yuval Bloch

**Affiliations:** ^1^BrainMARC LTD., Yokneam, Israel; ^2^Psychiatry Department, Emek Medical Center, Afula, Israel; ^3^Technion – Israel Institute of Technology, Haifa, Israel; ^4^Shalvata Mental Health Center, Hod Hasharon, Israel; ^5^Tel Aviv University, Tel Aviv, Israel

**Keywords:** depression, EEG, attention, prediction, treatment, brain engagement

## Abstract

**Introduction:**

The evaluation of response to pharmacological treatment in MDD requires 4–8 weeks. Therefore, the ability to predict response, and especially lack of response to treatment, as early as possible after treatment onset or change, is of prime significance. Many studies have demonstrated significant results regarding the ability to use EEG and ERP markers, including attention-associated markers such as P300, for early prediction of response to treatment. But these markers are derived from long EEG/ERP samples, often from multiple channels, which render them impractical for frequent sampling.

**Methods and results:**

We developed a new electrophysiological attention-associated marker from a single channel (two electrodes), using 1-min samples with auditory oddball stimuli. This work presents an initial evaluation of the ability to use this marker’s dynamics between repetitive measures for early (<2 weeks) differentiation between responders and non-responders to antidepressive treatment, in 26 patients with various levels of depression and heterogeneous treatment interventions. The slope of change in the marker between early consecutive samples was negative in the non-responders, but not in the responders. This differentiation was stronger for patients suffering from severe depression (*p* < 0.001).

**Conclusion:**

This pilot study supports the feasibility of the EEG marker for early recognition of treatment-resistant depression. If verified in large-scale prospective studies, it can contribute to research and clinical work.

## Introduction

The evaluation of response to pharmacological treatment in MDD requires 4–8 weeks, after which response rates are of the order of 50% and remission rate of 30% (1). Earlier and more specific identification of patients who are less likely to respond to a given treatment is therefore important both to improve our understanding of the underlying pathophysiology, and for daily clinical decision analysis (2). Suggested biomarkers include various clinical, neuroimaging, and genetic approaches (3). A biomarker of clinical significance is expected to be based on scientific understanding of the pathology, must have a sensitivity and specificity that contribute to the clinical work and must be applicable. Thus, we expect the biomarker to be accessible and to be useful in the complex and heterogeneous clinical settings.

Many studies have demonstrated significant abilities in using EEG and ERP markers based on pretreatment evaluation or on evaluation after several treatment days (4). Ample electrophysiological evidence has been accumulating regarding reduced attention in MDD. Reduced P300 amplitude, delayed latency, or both have often been reported (5–7). ERP attention markers, such as P300, are sensitive to the patient’s condition. When the condition improves, the waves tend toward the normalized amplitude and latency; the opposite occurs when the condition deteriorates (8, 9). Because attention is greatly affected by depression severity (10), attention-associated markers seem to be relevant to the clinical condition, regardless of the specific treatment and its mechanism of action (7). Thus, markers for attention accord with scientific understanding on one hand and may be relevant within the complex and heterogeneous environment of clinical practice on the other.

Some studies failed to demonstrate distinct attention-associated electrophysiological markers in depression (5). The markers are often based on samples of up to tens of minutes, either of raw EEG or of averaged ERP. But longer samples might reduce the precision of electrophysiological markers for attention. At least with regard to the ERP markers, there seems to be habituation of the attention response from the response to early blocks stimuli to that of subsequent ones. Furthermore, it seems that the degree of habituation correlates with the intensity of response in the early blocks (11, 12). Thus, the average ERP response between two samples may be indistinguishable, even if the response to early stimuli is significantly different because the stronger early response may habituate more, and the initial difference averages out over time. This blurring of differentiation may be prevented by effective analysis limited to the response to earlier stimuli.

In the last several years, we have developed an effective single-channel marker for attention. Our work shows that attention processes can be efficiently monitored over a wide frontocentral area, using prefrontal electrodes (13, 14). We developed a method to monitor prefrontal activity using a simple and minimal setup of two electrodes. We also simplified the EEG analysis to adjust the extraction of relevant attention-associated markers from a short sample of the scale of 1 min, based on template matching^1^^,^^2^ (15) of the marker identified by the averaged ERP. Template matching is the search in the sampled EEG data for a specific *a priori* pattern. We follow in this regard a known methodology, which scans the raw EEG data for patterns, which were identified in the averaged ERP signal (16). It should be stressed that while in the averaged ERP sample our marker is time locked to the stimulus (13, 14), we noted that the marker onset is much more flexible at the single-trial level. Such large temporal variability (in the scale of many hundreds of milliseconds) is known in the literature and is larger for the low frequency EEG activity. It was related with amplitude and phase of pre-stimulus oscillations (17). Due to this large flexibility, of hundreds of milliseconds, in evoked response latency we did not time-lock the template matching at the single-trial level.

We hypothesize that short, repetitive, simple EEG recordings during changes in pharmacotherapy can aid in the earlier detection of treatment resistance in the clinical complexity of different pharmacological changes during the treatment of MDD. We used template matching to extract attention-associated markers from 1-min long samples, to evaluate the dynamics after treatment change and to distinguish early between MDD responders and non-responders. The use of only the first minute of the sample is consistent with the above suggestion to focus on the electrophysiological response in the initial sample blocks. It was shown in various experimental designs that there is greater habituation when the initial response is stronger (11, 12). This differential habituation can average out initial differences, and therefore there might be an advantage to the analysis of the difference between groups of the initial responses, or in our case the first minute of response to stimuli.

In this initial study, we sought to test the generality of the above hypothesis and therefore included patients with heterogeneous pharmacological treatment changes.

## Materials and Methods

### Participants

Patients were recruited from two different settings: an outpatient psychiatric clinic of a general hospital (psychiatric department of the Emek Medical Center, Afula, Israel), and the outpatient clinics of a mental health center (Shalvata Mental Health Center, Hod Hasharon, Israel). Thirty MDD patients and 10 control subjects were included in the study. Of these, 26 patients and the 10 controls completed the study, which involved 14–16 sampling meetings over a period of 2–3 months. Four patients dropped out during the first weeks and were not included in further analyses. Three of these patients did not comply with the requirement for repeated sampling and withdrew in the first 2 weeks. The fourth patient was excluded after about 2 weeks of sampling because it was found that, along with the pharmacological treatment change, he also started electroconvulsive treatment (ECT), which affects EEG significantly (18). Participants were recruited through the practicing physicians in the two medical centers. The principal inclusion criterion for the MDD patients group was change in pharmacological treatment in the 2 days preceding the first study sample for any consideration (clinical deterioration, side effects, etc.). Any common pharmacological strategy intervention was accepted, including switch from one drug to another, augmentation, combination, or change in the dosage of an ongoing treatment. Most often, the change was the result of clinical deterioration or lack of response to preceding treatment. In several patients, the change in treatment was intended to reduce side effects or redundant treatment. Additional inclusion criteria for both the patient and the control groups were as follows: (a) age of 18–80 years and (b) ability and willingness to comply with study requirements of one to two sampling meetings each week. Exclusion criteria for both groups were as follows: (a) diagnosis of psychotic disorder, (b) diagnosis of any neurological disorder, (c) use of recreational or illicit drugs, or a recent history of drug or alcohol abuse or dependence, (d) hearing disorder, and (e) high risk of suicide as evaluated by the study PIs. A specific inclusion criterion for the controls group was a low grade (<2.3) in the Brief Symptom Inventory (BSI). Note that severity of depression, as measured by Hamilton Rating Scale for Depression 21 (HamD), was not a criterion for inclusion or exclusion. Note further that neither bipolar disorder nor level of anxiety excluded patients from participating in the study. Nevertheless, no bipolar patients were eventually recruited, and only two recruited patients suffered also from severe comorbid anxiety (HamA ≥ 25). The study was approved by the local ethics committee at both centers. After receiving a general explanation from their physician, patients were referred to the study. Patients then completed the informed consent process with the study physicians, where it was stressed that clinical decisions and therapy were not affected by participation in the study.

As we expected based on our attempt to monitor the diversity of “real-life” clinical practice, patients differed in age, gender, type, and reason for pharmacological change, and the outcome of this change (Table 1). For our analysis, patients were grouped according to depression severity as evaluated by HamD in the first week. Thirteen patients suffered from severe depression, with an HamD score of 19 or above, 9 were evaluated as suffering from mild to moderate depressive episode (HamD of 10–19), all 10 controls as well as an additional 4 patients had an HamD of less than 7 (the reason for the patients’ pharmacological change was side effects). The analysis of outcome was based on improvement in HamD score between the first to the last sample weeks. Seven participants in the severe depression group and five participants in the mild–moderate group were defined as responders, based on at least 50% improvement in HamD; four of the severe and three of the mild–moderate were classified as non-responders, defined as improvement of less than 25% in HamD (19), including a deterioration in HamD, and two among the severe depression and one among the mild–moderate group were partial responders, defined as improvement of between 25 and 50% in HamD.

**Table 1:** Patients characteristics: demographics, clinical condition and changes in pharmacological treatment.

Patient#	Age	M/F	HamD—first week	HamD—last week	% Change	Drug	Change	Dosage (mg)
1	53	F	27.5	24.5	11	Venlafaxine	Start	150
						Olanzapine	Increase	5 to >10
2	66	F	27	26.5	2	Milnacipran	Start	50
						Venlafaxine	Decrease	150 to >75
3	55	F	27	16.5	39	Sertraline	Start	200
						Sulpiride	Start	50
						Aripiprazole	Start	5
4	55	F	25.5	15.5	39	Fluoxetine	Start	20
5	55	F	25.5	11	57	Fluvoxamine	Start	100
6	24	M	25.5	11	57	Sertraline	Start	50
7	23	F	25.5	7	73	Quetiapine	Start	150
						Trazodone	Increase	100 to >150
8	61	M	24	19	21	Vortioxetine	Start	10
						Bupropion	Start	150
9	71	F	23	5	78	Sertraline	Start	50
10	63	F	21	7.5	64	Quetiapine	Start	50
11	23	F	20	9.5	53	Sertraline	Start	50
12	19	F	19	18	5	Escitalopram	Start	10
13	50	F	19	6	68	Bupropion	Start	150
14	48	F	16.5	25	−52	Quetiapine	Start	50
						Sertraline	Start	50
15	26	M	16.5	3.5	79	Sertraline	Start	100
16	50	F	16	16	0	Venlafaxine	Increase	150 to >225
17	47	M	15	2	87	Mirtazapine	Start	15
18	21	F	13	2	85	Quetiapine	Increase	150 to >300
						Bupropion	Stop	150 (–)
						Perphenazine	Stop	8 (–)
						Biperiden	Stop	12 (–)
19	21	M	11.5	5	57	Venlafaxine	Start	150
						Fluvoxamine	Decrease	200 to >150
20	51	F	11	6	45	Venlafaxine	Decrease	225 to >150
21	61	F	11	2	82	Venlafaxine	Start	150
22	56	F	10	16	−60	Bupropion	Start	150
23	66	M	7	2	71	Sertraline	Increase	50 to >100
24	74	F	6	6.5	−8	Venlafaxine	Start	150
						Quetiapine	Start	100
						Escitalopram	Stop	5
25	66	F	2.5	3.5	−40	Venlafaxine	Decrease	225 to > 150
26	65	M	0	2.5		Venlafaxine	Decrease	150 to >75

### Procedure

After completing the informed consent process, patients underwent basic evaluation to verify that they meet inclusion criteria. After inclusion in the study, all participants in both groups underwent 13–16 EEG sampling sessions over a period of 2–3 months. The EEG data were recorded from the NeuroSky MindWave single-channel system (NeuroSky Inc., San Jose, CA, USA—CE authorized), with one frontal electrode (~Fpz) and one reference electrode on the earlobe, at a sampling rate of 512 Hz. In previous works, we noted that our template marker could be extracted from any sagittal or parasagittal electrode in the central and frontal regions, if the reference is periauricular (14). As a setup of dry electrodes, which sample below the hairline is easier to use, we chose the Fpz frontal location and the earlobe reference location. The MindWave EEG headset uses dry EEG electrodes. The sampled data were transferred through a wireless connection to the experiment computer for offline processing. Each sampling session involved 5 min of stimulus-free recording and 5 min of recording that was synchronized with an auditory oddball protocol. The data from the 5-min stimulus-free sample were not analyzed in this study. Nevertheless, we cannot exclude a possible influence (e.g., adjustment to sampling device), of this passive period, on the sampled data from the succeeding sampling period, which involved the auditory oddball stimuli. The oddball stimuli consisted of 1,000 and 2,000 Hz pure tones of 40 ms duration, presented binaurally at ~60 dB using earphones. The stimuli were comprised of a frequent tone (1,000 Hz) presented 80% of the time, and a rare tone (2,000 Hz) presented 20% of the time. Interstimulus interval was selected randomly in the range of 2–3 s. We followed a conventional unbalanced oddball protocol (20–80%) with the aim of reducing habituation (20). The participants were instructed to listen passively to the stimuli, without responding (21). Only data from the first minute of the stimulus-related samples were used in the analysis. The role of the oddball paradigm was to maintain a higher level of attention, as described in the literature in task-related analysis studies (22). We did not differentiate between the two types of stimuli in the data analysis.

All patients also underwent evaluation with the 21-item Hamilton depression rating scale (HamD) once every 7–10 days, on the days of their sampling sessions. The sampling sessions and HamD evaluations took place at the clinics or at the patients’ home, at the patients’ convenience. The settings of the sampling sessions were the same in repetitive meetings for each patient. The first and last HamD evaluations were conducted by the research physician. All other HamD evaluations were conducted by research assistants, who underwent guidance regarding the proper use of the scale. The raters of the HamD were blind to the results of the EEG evaluation.

### Data Analysis

#### Clinical Data Analysis

Depression severity and treatment response were evaluated using the HamD (23) for each participant. The initial HamD value was the average of the two HamD evaluation scores in the first sampling week. If the difference between the first two scores was greater than 6 points, the median of the first three evaluations was used as the baseline score. Similarly, the final HamD value was the average of the two HamD evaluations in the last sampling week. If the difference between the two last week evaluations was greater than 6, the median of the last three HamD evaluations was used as the final HamD value. Average weekly HamD values were also computed for each patient to evaluate the dynamics. Early response was also evaluated weekly and compared with the HamD of the first week. A threshold of 20% improvement was used to determine early response (2).

#### Brain Engagement Index (BEI) Analysis

**Figure 1:**
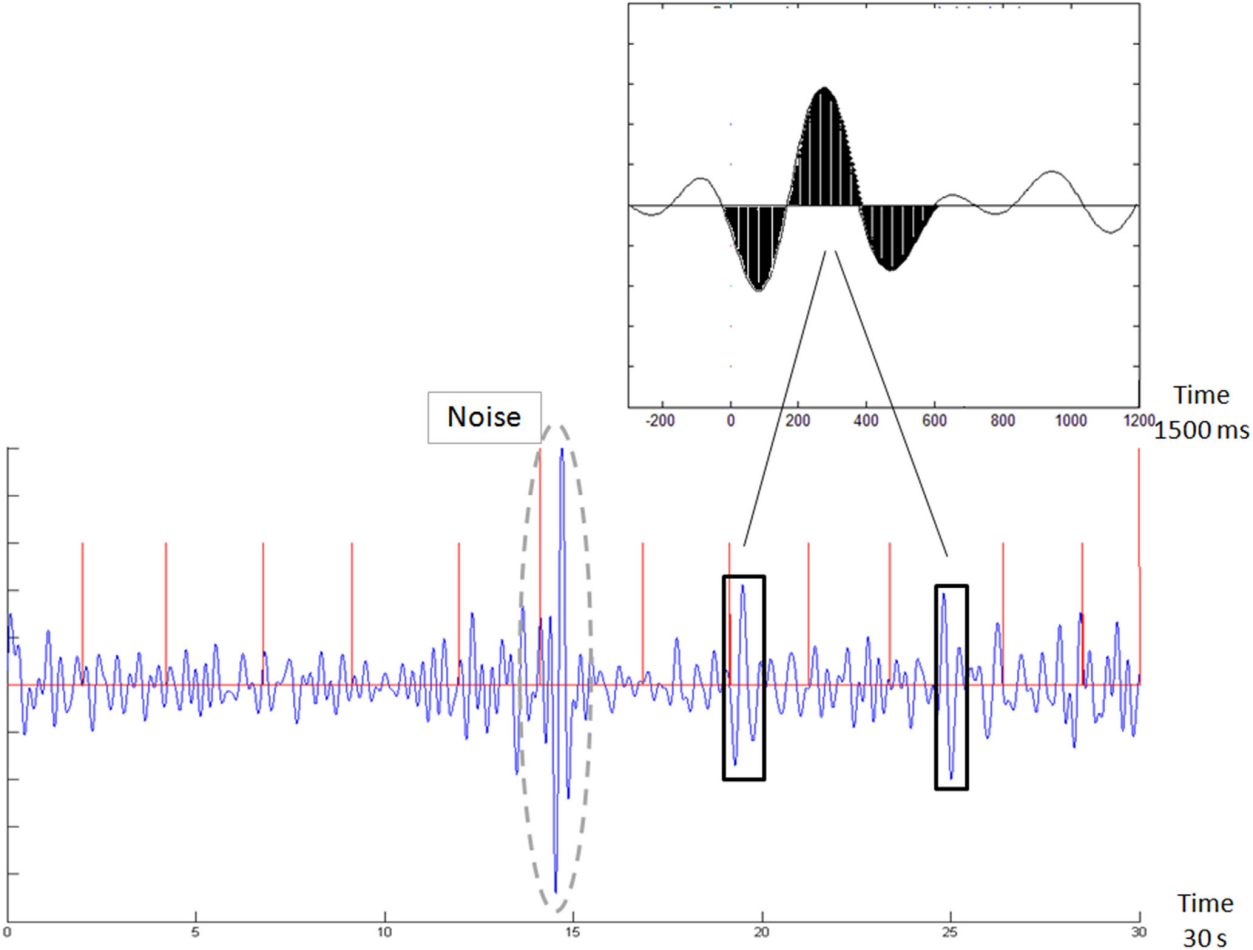
Demonstration of template matching. The template is emphasized in black in the top inset. The new sample in the bottom figure is scanned with a moving window, following normalization to the [−1, 1] range. Whenever a match is found (in black rectangles), it is counted. The Brain Engagement Index is a normalization of this count to the [0, 1] range. The bottom figure also shows an automatically rejected noisy sample (surrounded by a dashed gray line). Stimulus times are marked with red vertical lines.

We computed the Brain Engagement Index for data from the first minute of the stimulus-related sample. The computation is based on template matching (14), a technique that uses a basic template, which is compared with the sampled signal. In this case, the template was a 1,500 ms attention-associated averaged ERP delta bandpass activity (14), which was matched with a moving window of the same size in the sampled signal. The matching was performed as follows: (i) the 1-min sample was divided into segments of 10 s; (ii) each segment was filtered in the delta bandpass (1–4 Hz); (iii) the data points in the filtered segment were normalized to the [−1, +1] range, where −1 denotes the most negative and +1 the most positive voltage deflection within the filtered segment; (iv) the process of filtering and normalization to [−1, +1] was also performed for the 1,500 averaged Delta ERP wave, shown in Figure 1 top inset, to generate the template [taken from Ref. (14)]; (v) the normalized sampled segment was scanned by a moving window of 1,500 ms, with 1 ms moving steps; (vi) Noise rejection: for every 1,500 ms window, we also computed the SD mean ratio.

Based on our manual inspection and our accepted method of noise rejection (24), if this ratio is greater than 1, the sampling is likely to be noisy, and therefore these 1,500 ms samples were rejected and not included in the above computation. If multiple non-overlapping 1,500 ms windows were rejected within a given 10-s segment, the entire segment was automatically rejected. At least three consecutive 10-s segments were required to be valid to generate a valid BEI for the entire sample. Otherwise, the entire sample was rejected as noisy and was excluded from the next steps of analysis. If the samples of the first day or 2 days were rejected as noisy, participants were instructed to close their eyes in consequent samples. Five participants were instructed after one to two samples to perform the samples with closed eyes. The spread of these participants was rather even across study groups was rather even (one responder, one non-responder, one euthymic patient, and two control participants). All in all, about 40% of the samples were rejected as noisy, spreading rather evenly in the various clinical groups. (vii) The averaged distance between the moving window data and both the template and the template opposite (negation of template) were computed; (viii) if the averaged distance was less than a threshold (0.5 for the template or template opposite, as shown in Figure 1), the match count was increased—provided that no other match was found in a previous window, partly overlapping with the current one; (ix) if the averaged distance was more than the threshold, the no-match count was increased—provided that no other no-match was found in a previous overlapping window; (x) the BEI is the ratio of the match count to the no-match count (maximum BEI value was set to +1, with BEI scale of [0, 1]).

Based on large single-trial variability (25), the matching was not locked in time with the stimuli. This single-trial variability might be especially noticeable for the slow wave activity of the delta band used in this study and is related with pre-stimulus oscillation amplitude and phase (17). We noted that for certain trials, the activity tends to increase immediately after stimulus, while in other trials it decreases thereof and increases with a delay of few hundred milliseconds, probably in relation to the level of baseline activity preceding the stimulus. Furthermore, we made no distinction between the two types of stimuli.

#### Analysis of Dynamics in BEI

After computing the BEI for the single samples, we computed dynamics indices between consecutive samples. The elementary dynamics indices were the average weekly and biweekly BEI values (for weeks 1 and 2, weeks 3 and 4, weeks 5 and 6, and weeks 7 and 8), which were computed for each participant.

**Figure 2:**
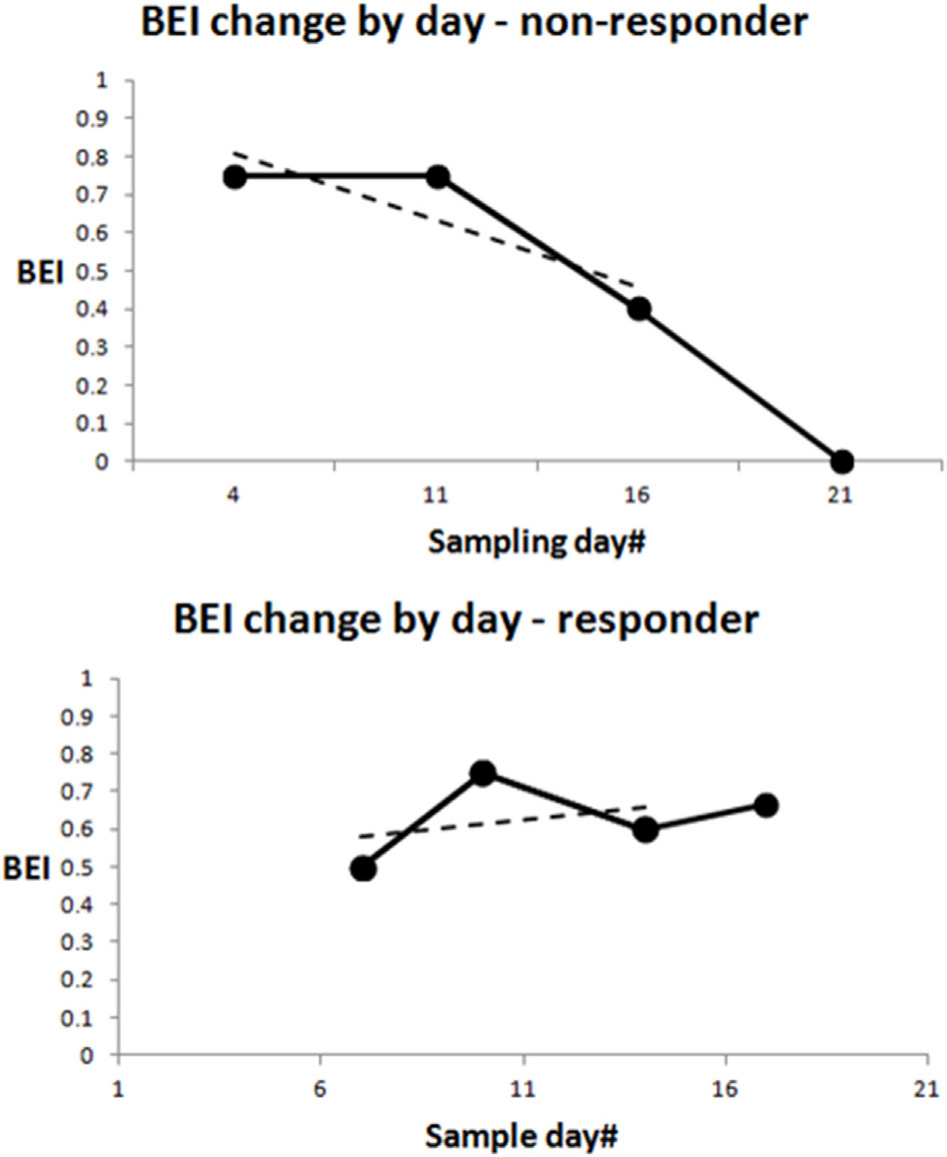
Demonstration of Brain Engagement Index (BEI) dynamics. Data from two patients. (i) The solid black line shows the dynamics between the first four sampling points. If the dynamics are monotonically non-increasing, with a total drop of at least 0.2 between the first and fourth BEI in the sequence, a BEI drop is determined. The upper graph shows a BEI drop. Note that BEI drops could start with any sample in the first month, not necessarily with the first sample, as presented in the graph. The only requirement is the occurrence of a drop in four consecutive samples. Valid BEI samples are interspersed by invalid (noisy) ones, which are not included in the BEI drop evaluation. (ii) The dashed line represents the linear slope between the first three sampling points. The slope index is computed for participants with at least three valid BEI values in the first three sampling weeks. It is the slope of a linear regression between the first three valid BEI values sampled for the participant. We did not compute a slope index for participants with fewer than three valid BEI values in the first three sampling weeks.

The binary *BEI drop index* is positive if any four valid (non-noisy) BEI values of consecutive samples are monotonically non-increasing (they either decrease or stay the same), and if there is a BEI drop of at least 0.2 from the first to the last value in the set of four values. *The timing of the BEI drop index* was determined as the week in which the fourth value in the set was sampled. If no BEI drop was found, with a starting sample in the first sampling month (the first value in the set of four was sampled in the first month), the BEI drop index for this participant was negative. Figure 2 (top) shows a participant with a BEI drop already in the first four valid samples (solid black line), and at the bottom, samples from a participant with no BEI drop in at least the first four samples. Note that the valid BEI samples could be interspersed with invalid (noisy) ones, which are not included in the BEI drop evaluation.

We computed the *slope index* for participants with at least three valid BEI values in the first three sampling weeks. It is the slope of a linear regression between the first three valid BEI values sampled for the participant. We did not compute a slope index for participants with fewer than three valid BEI values in the first three sampling weeks. Figure 2 shows the slopes of the two participants presented (dashed lines).

#### Statistical Analysis

The evaluation of the BEI change between weeks 1 and 2 and weeks 7 and 8 was performed for the groups of severely depressed (initial HamD ≥ 19) and all-depressed patients (initial HamD ≥ 10). Note that the group of all-depressed patients also includes the severely depressed group. The evaluation was performed using a two-way ANOVA, with independent response (responders vs. non-responders) and time (weeks 1 and 2 vs. weeks 7 and 8) variables. We further evaluated the difference in occurrence of BEI drops between responders and non-responders in both the severely depressed and all-depressed groups using chi-square tests. Finally, we evaluated the difference in the slope index between responders and non-responders in both the severely depressed and all-depressed groups using *t*-tests.

## Results

### Clinical Outcome

Twenty six patients (19 females, 7 males, 48.85 ± 17.75 years old) and 10 controls (6 females, 4 males, 47.30 ± 21.73 years old) were included in the study. Thirteen of the patients suffered from severe depressive episodes (HamD ≥ 19) at the beginning of sampling. Seven patients from the severely depressed group responded to treatment, four did not respond to treatment, and two responded partially to treatment. Twenty-two participants suffered from active depression (HamD ≥ 10, including the severe depression patients described above). Of this all-depressed group, 12 patients responded to treatment, 7 did not, and 3 responded partially. Four patients with a history of depression and active antidepressive treatment, and 10 control participants were euthymic at the beginning of sampling.

### Dynamics of BEI As a Function of Depression Severity and Response to Treatment

**Figure 3:**
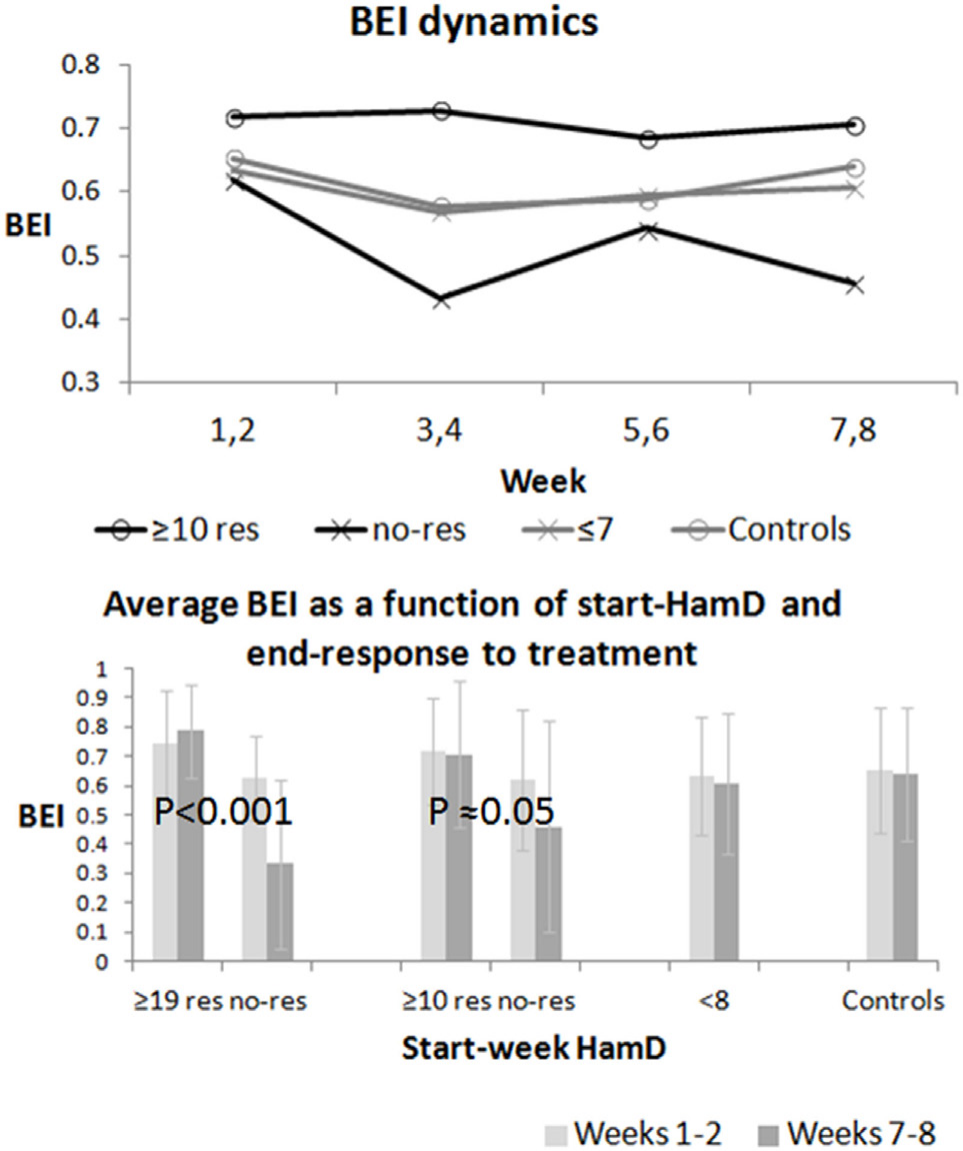
Global Brain Engagement Index (BEI) dynamics. The top graph presents the average BEI (over all participants) for every two sampling weeks in the all-depressed responding and non-responding patient groups, and in the two control groups (controls and euthymic patients). The bottom chart shows the change in the average BEI between weeks 1 and 2 and weeks 7 and 8. In this figure, the change is also shown separately for severely depressed patients (responders vs. non-responders). The BEI decreases between weeks 1 and 2 and weeks 7 and 8 in the non-responding groups, but not in the responding groups, and the difference is statistically significant both for the severely depressed and the all-depressed patient groups.

Figure 3 shows the dynamics of BEI over the sampling period. The top graph shows the average biweekly BEI value for all participants in four groups: all-depressed (HamD ≥ 10), who responded to treatment change, all-depressed who did not respond to treatment, euthymic patients (HamD ≤ 7), and controls. The bottom graph shows the average and SD of the BEI in weeks 1 and 2 and 7 and 8 in all these groups as well as in severely depressed patients (HamD ≥ 19), who either did or did not respond to treatment intervention. A two-way ANOVA computed for the severely depressed patients revealed significant differences between outcome groups {responders vs. non-responders [*F*(1,16) ≈ 14.3; *p* ≈ 0.001]}, but not between weeks 1 and 2 and 7 and 8 [*F*(1,16) ≈ 0.34; *p* ≈ 0.57]. We found an interaction between outcome groups and time [*F*(1,16) ≈ 8.17; *p* ≈ 0.01]. A two-way ANOVA computed for the all-depressed patients did not reveal significant differences between outcome groups {responders vs. non-responders [*F*(1,32) = 2; *p* ≈ 0.17] or time [*F*(1,32) ≈ 0.25; *p* ≈ 0.62]}. We found an interaction between outcome groups and weeks [*F*(1,16) = 4; *p* ≈ 0.05]. After excluding the samples of the patients, who were instructed to close their eyes due to noisy first samples, two-way ANOVA revealed similarly significant differences between the severely depressed responders vs. non-responders [*F*(1,14) ≈ 9.86; *p* < 0.01], but not between weeks 1 and 2 and 7 and 8 [*F*(1,14) ≈ 0.32; *p* ≈ 0.58]. We again found an interaction between outcome groups and time [*F*(1,14) ≈ 7.95; *p* ≈ 0.01]. A similar two-way ANOVA of the remaining (opened eyes) samples for the all-depressed patients did not reveal again significant differences between outcome groups {responders vs. non-responders [*F*(1,28) = 0.72; *p* ≈ 0.40]} or time [*F*(1,28) = 0.24; *p* ≈ 0.63]. The interaction between outcome groups and weeks did not reach significance [*F*(1,28) = 3.14; *p* ≈ 0.09], possibly due to the reduction of sample size. Significant interaction, which was more evident for the severely depressed, means that BEI remains high for the responding patients, but decreases for the non-responding ones.

### Continuous Drops by Clinical Group and Response to Treatment

**Figure 4:**
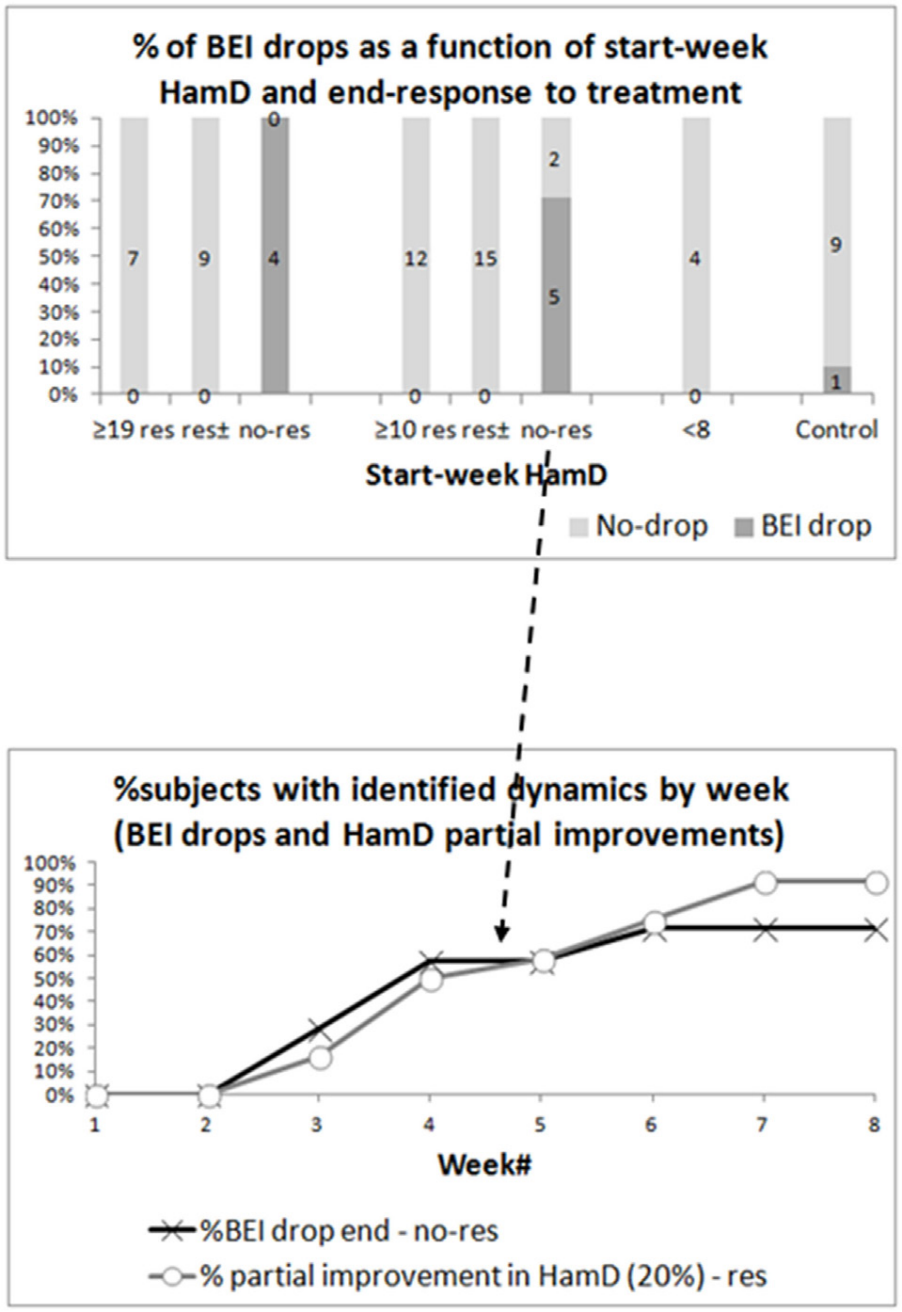
Brain Engagement Index (BEI) drops. The top graph presents the percentage of drops in BEI between four consecutive samples, starting at any point during the first sampling month, in the various groups. A BEI drop is a monotonic non-increasing dynamics between four points, with a reduction of at least 0.2 in the BEI between the first and fourth samples. The percentages are shown for the severely depressed patients [divided into responders (res), responders + partial responders (res±), and non-responders (no-res)], all-depressed patients (divided into responders, responders + partial responders, and non-responders), euthymic patients, and controls. For both depressed groups, the difference between responders and non-responders was statistically significant. The bottom graph shows the week in which the fourth sample point of the BEI drop was taken in all-depressed non-responders (black arrow). It reached ~71%, as five of seven non-responding patients demonstrated a drop in BEI. For comparison, it also presents the week in which at least 20% improvement in HamD was first noted in responders.

The top graph in Figure 4 shows the percentage of participants who demonstrated a drop in BEI starting from the first sampling month. The subgroups presented are the severely depressed responding and non-responding patients, the all-depressed responding and non-responding patients, the euthymic patients, and the controls. To differentiate the non-responding patients from responders and partial responders, we added another subgroup (res±), which includes both responders and partial responders among both the severely depressed and the all-depressed patients. We used chi-square tests to compare the occurrence of BEI in responding and non-responding groups, and results were significant for both the severely depressed (*p* < 0.001) and all-depressed patients (*p* < 0.001). The difference in BEI drop occurrences remained significant after excluding the samples of the patients, who were instructed to close their eyes due to noisy samples (severely depressed—*p* < 0.001, all-depressed, *p* < 0.001).

The bottom graph in Figure 4 shows the percentage of participants from the all-depressed non-responding group who completed the four-sample BEI drop sequence, by week of sampling (solid black line). Note that only five out of seven non-responding patients demonstrated a BEI drop, therefore the maximum percentage of participants by week is ~71%. For the sake of comparison, the gray line presents the percentage of responding patients, who demonstrated at least 20% early improvement in HamD score by week.

### Early Tendencies by Clinical Group and Response to Treatment

**Figure 5:**
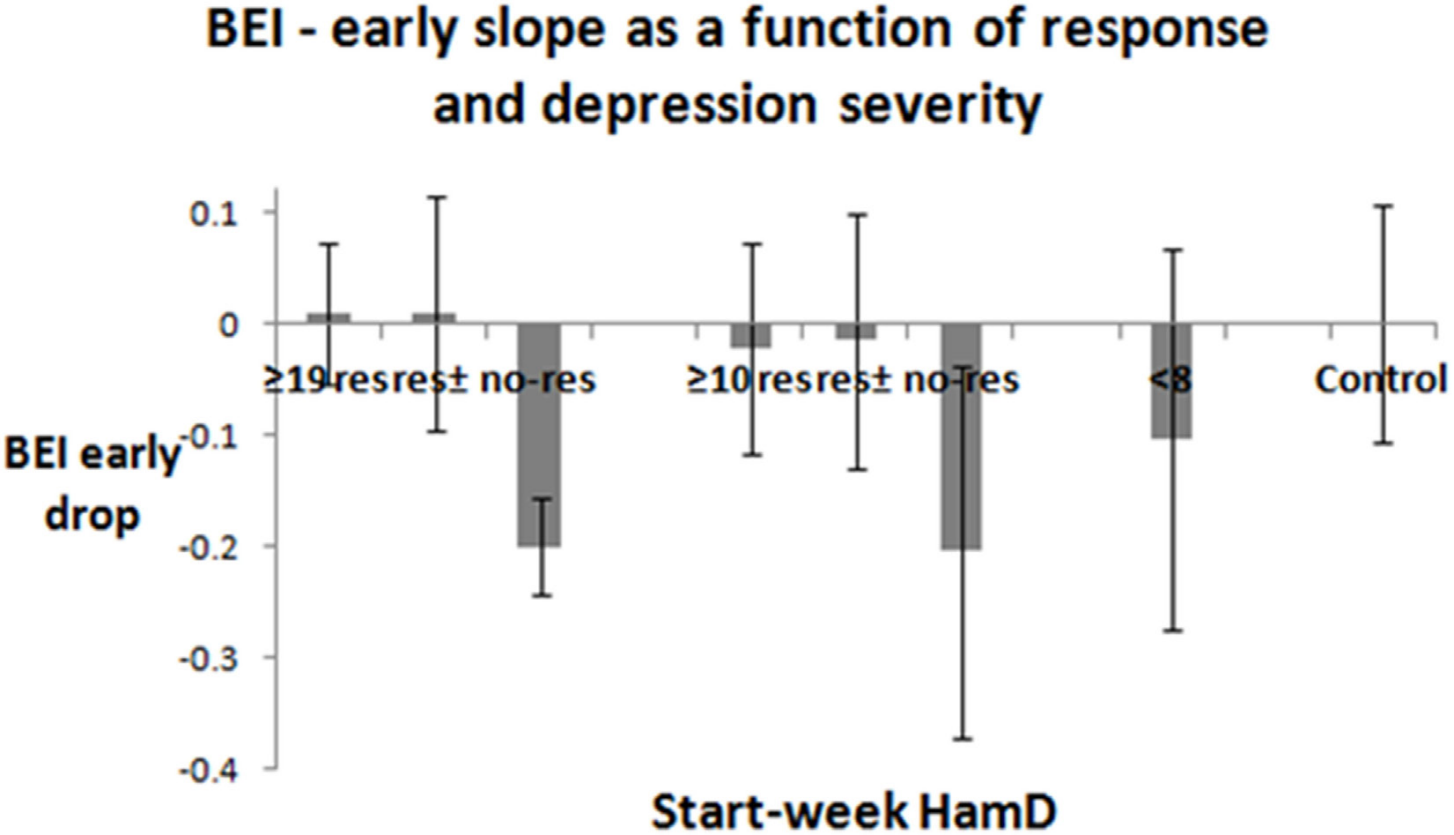
Early slopes. In 10/13 of the severely depressed patients, and in 16/22 of all-depressed patients, there were at least three valid sample points in the first 3 weeks. The linear slope between the first three valid sampling points was evaluated. Its average and SD are presented for the responders, partial responders, and non-responders among the severely depressed and all-depressed patient groups. It is also shown for the depressed participants during remission and for the control groups. Lack of responsiveness to treatment change at the end of the sampling period (2–3 months) was significantly associated with a negative slope. The three sampling days were completed in 12.74 ± 3.56 calendar days (mean + SD).

Figure 5 presents the average and SDs of the linear slopes of the participants in the different groups. The graph presents the bars of the following groups: severely depressed (responders, responders + partial responders, non-responders), all-depressed (responders, responders + partial responders, non-responders), euthymic patients, controls. *t*-Tests were significant, when comparing the slopes of the responders and non-responders for both severely depressed patients (*p* < 0.001) and all-depressed patients (*p* ≈ 0.02). The slope differences remained significant after excluding the samples of the patients, who were instructed to close their eyes due to noisy samples (severely depressed—*p* < 0.001, all-depressed, *p* < 0.05). Note that 10 out of 13 patients in the severely depressed group, and 16 out of 22 in the all-depressed group had at least 3 valid sampling points in the first 3 sampling weeks, and therefore it was possible to compute slopes for them. For all-depressed patients, the third valid point (and therefore the slope) was sampled in 12.74 ± 3.56 days. An initial slope below −0.1 predicted no-response in 83% of the cases. An initial slope above −0.1 predicted response in 86% of the cases.

## Discussion

The present pilot study supports the use of repeated EEG monitoring as a biomarker of resistance to antidepressant pharmacotherapy. The monitoring was made feasible by the basic equipment used and a simple to imply algorithm based analysis. Almost 90% of patients of two outpatient public clinics were able to complete the repetitive samples, and the 60% of valid samples were adequate for the analysis. In the presented study, an early (~12 days) drop in the BEI of a depressed patient following a treatment change correlated with an 8-week non-response, regardless of the specifics of the treatment intervention. This was true especially among the severe depression group, which was consistent with our pathophysiological expectations (10).

Note that this is an initial evaluation study. We sampled heterogenic depressed patients who underwent pharmacological antidepressive intervention mostly due to lack of response to previous treatment, but also due to a desire to reduce or ameliorate side effects. The patients were heterogenic both in the severity of their depression symptoms and with regard to the pharmacological treatment strategy. A heterogenic study of this type has its obvious pitfalls, especially if one considers the different expected mechanisms by which various treatments affect the brain. Nevertheless, the BEI, a marker used for attention, may be affected by various pharmacological interventions, regardless of their precise mechanism of action (7). Therefore, the BEI represents a common pathway of influence that may be considered independent of the specific mechanism of action of vast antidepressive intervention. The motivation to include in this pilot study patients with various degrees of clinical severity, and various types of treatment changes, was to obtain an initial impression regarding the robustness on the dynamics of the BEI for real-life settings. Nevertheless, it is obvious that in future studies, it would be of much value to evaluate the dynamics of the BEI in much larger and more homogeneous patient groups.

We noted that, despite the heterogeneity of treatment interventions, the BEI tended to decrease in non-responders and to maintain high levels in responders (Figure 3 and related text). We further demonstrated that the change in BEI in non-responders took place within a few weeks after treatment change (Figure 4 and related text), within a timeframe comparable to the early changes in HamD score in responders. Thus, the BEI can contribute complementary information for early treatment change, with the potential to increase the specificity of early evaluation, which is currently limited (2). Furthermore, we demonstrated that even within the first 2 weeks of treatment, the BEI slope of non-responders was significantly more negative than that of responders and of partial responders, making an even earlier evaluation possible. The aforementioned trend was even more robust in severely depressed patients. Note that the control group and the small euthymic group (which may be viewed as another designated control group) tended to maintain stable BEI over time, without the dropping, which characterizes non-responsive patients.

The phenomenon of BEI drop in the non-responders is intriguing. The majority of these patients did not deteriorate clinically, but rather failed to improve. Our experience with the BEI (see text footnotes 1 and 2) shows that it tends to be high in the first sample(s) in certain patient groups, especially when comorbid anxiety is involved. This possibly stems from the novelty of the sampling situation, which may recruit more attention. Such a phenomenon might be prominent in depressed patients with a comorbidity of anxiety. In such patients, the initial BEI is high, but then decreases if there is no change in the clinical state. At the same time, if there is clinical improvement, which also involves improved attention, the BEI does not decrease. Note that more than differences in baseline BEI, it is the dynamics in BEI in the course of antidepressive intervention that distinguishes the non-responders from the other groups. The dynamics may be stronger for severely depressed patients because they reach lower BEI values faster.

The conclusions that can be derived from this initial evaluation study are limited by the small sample size, the variability in the initial patient conditions, a placebo effect that may have emerged from intensive sampling, and by the heterogeneity of the treatment changes. Much larger and possibly more homogeneous studies need to be conducted to support the conclusions derived from the present study. An appealing attribute of this study is that its underlying technology is extremely easy to use, even at home by the patient, as it involves a simple 2-electrode setup and a 1-min sample. Of potential practical value is the use of dry electrodes below the hairline—Fpz referenced to earlobe. We showed previously the feasibility of extracting significant markers from this region (14). Obviously sampling from the forehead is always susceptible to noise, especially due to eye movements, but with the use of effective noise rejection method (24) together with the instruction to close the eyes, when the noise was too large, we managed to obtain an effective marker. It nevertheless seems of significance to evaluate the effect of eye closing on the BEI marker in a systematic manner with a larger study. The other potential practical value is the use of short sample. The ability to move from averaged ERP to raw EEG by template matching enabled us to harness the long standing observation that differentiation between groups might be more prominent with a shorter sample. This is because there is apparently greater habituation of stronger initial responses, which seems to average out differences (11, 12). Note further that the study was based on one or two samples per week. Sampling more frequently may reduce the time needed to detect the dynamics of the BEI.

## Ethics Statement

The study was approved by the ethics committees of Emek Medical Center and of Shalvata Mental Health Center. All participants signed an informed consent after detailed explanation.

## Author Contributions

GS, YB, and SY designed the study. GS developed the BEI index and analyzed the data. YB and SY served as PIs. BB, UN, AS, and AR helped in the study design and in patient recruitment and clinical evaluation.

## Conflict of Interest Statement

GS is the co-founder and chief scientist of BrainMARC LTD (the developer of the BEI marker used in this study). YB and SY served as PIs in the study. The other authors did not have a conflict of interest.

## Footnotes

^1^Bartur G, Joubran K, Peleg-Shani S, Vatine JJ, Shahaf G. Engagement, its relevance to stroke recovery and neurorehabilitation: an applicative on-line EEG tool for enhancing treatment efficacy. (2017).

^2^Shahaf G, Kuperman P, Bloch Y, Yariv S, Granovsky Y. Monitoring migraine cycle dynamics with an easy-to-use electrophysiological marker. (2017).
